# Case report: Two novel *intergenic region-ALK* fusions in non-small-cell lung cancer resistant to alectinib: A report of two cases

**DOI:** 10.3389/fonc.2022.916315

**Published:** 2022-07-22

**Authors:** Shan Liao, Huiying Sun, Jianhua Wu, Hao Lu, Yisheng Fang, Yuanyuan Wang, Wangjun Liao

**Affiliations:** Department of Oncology, Nanfang Hospital, Southern Medical University, Guangzhou, China

**Keywords:** non-small-cell lung cancer, *intergenic region-ALK* fusion, next-generation-sequencing, alectinib, chemotherapy, anti-angiogenesis therapy

## Abstract

**Background:**

The anaplastic lymphoma kinase (*ALK*) mutation, also known as the diamond mutation in non–small-cell lung cancer (NSCLC), has been treated with tremendous success since it was first reported in 2007. Alectinib, a second generation ALK-Tyrosine kinase inhibitor (TKI), has been reported to have significantly longer progression- free survival (PFS) than first generation ALK inhibitors in untreated ALK positive NSCLC. However, the clinical efficacy of ALK-TKIs on rare ALK fusions remains unclear. In recent years, with the popularity of next-generation sequencing (NGS) technology, an increasing number of novel ALK fusion partners have been reported, but the responses are heterogeneous among different ALK fusions. Considering the inconsistent reactions, the clinical efficacy of ALK-TKIs in rare ALK gene fusions remains to be evaluated in more cases.

**Methods:**

To seek for individualized therapy, the tumor tissues acquired during biopsy were sent for genomic testing by NGS based on a 139-gene panel and a 425-gene panel in a centralized clinical testing center (GENESEEQ Technology Inc, Nanjing, China). See **Supplementary Material** for more details about the methods for DNA-based NGS, RNA-based NGS.

**Results:**

We present two cases of patients with lung adenocarcinoma harboring two novel *Intergenic Region* (*IGR*)*-ALK* rearrangements detected by DNA sequencing, which had limited clinical response to ALK-TKIs but showed sensitivity to chemotherapy combined with bevacizumab therapy in patient 2, with a PFS of over 1 year up till the last follow‐up assessment.

**Conclusions:**

In summary, our cases emphasize the need for comprehensive molecular analysis of different ALK fusion partners at the DNA level to formulate accurate treatment strategies and provide a certain therapeutic reference for these two types of novel *IGR-ALK* fusions.

## Introduction

*ALK* gene rearrangement is a common driving oncogene in patients with NSCLC, approximately 3%-5% of non-squamous NSCLC harbor an *ALK* rearrangement, predominantly in younger, female patients who are light- or non-smokers (1–3). Echinoderm microtubule-associated protein-like 4 (*EML4*) is the most common fusion partner gene (3). Alectinib is a competitive ATP inhibitor of ALK tyrosine kinases, which can inhibit the phosphorylation process of ALK and the activation of downstream signal proteins STAT3 and AKT mediated by ALK, thus reducing the activity of tumor cells and finally promoting apoptosis in tumor cells (4). Updated data from the ALEX study showed that alectinib historically extended the median PFS in ALK-positive NSCLC to 34.8 months (5). However, the ALK status of patients enrolled in the ALEX clinical trial was confirmed only by immunohistochemistry (IHC), and detailed information about ALK fusion partners was not provided. Thus, whether alectinib shows comparable efficacy in patients with rare ALK fusion partners, remains to be further studied. Through the previous literature and case reports, more than 90 fusion partners have been identified in ALK-positive NSCLC (6). To note, *IGR-ALK* fusions are rarely reported, and their response to ALK-TKIs remains unclear (6, 7). Our cases have expanded the genomic landscape of ALK fusions and provide valuable clinical evidence for the treatment of patients with these two types of *IGR-ALK* fusions.

## Case presentation

### Case 1

**Figure 1:**
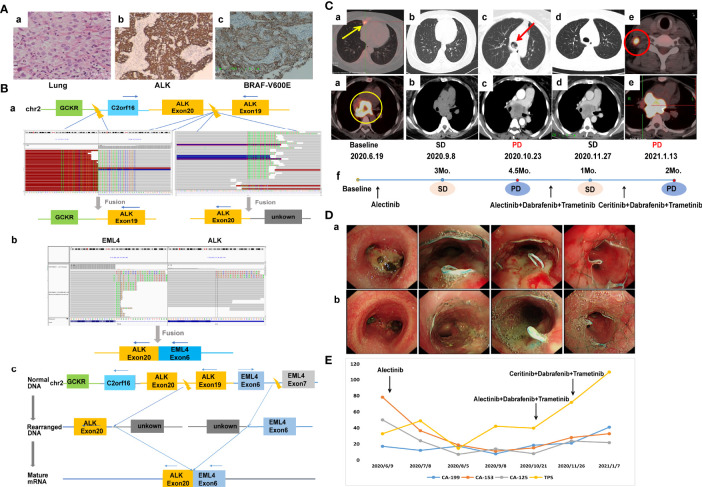
Case 1. **(A)** Pathology results. **(a)** H&E staining shows lung adenocarcinoma (40×). **(b, c)** IHC shows positive expression of ALK and BRAF-V600E after alectinib resistance (40×). **(B)** Identification of the fusion sequence by NGS. **(a)** The identification of *GCKR*-*ALK* and unknown-*ALK* fusion by DNA sequencing, visualized by the Integrative Genomics Viewer (IGV). **(b)** The identification of *EML4*-*ALK* by RNA sequencing. **(c)** Schematic diagram illustrating molecular rearrangements that explain the emergence of the *EML4* and *ALK* mRNA based on the DNA and RNA NGS results. **(C)** Radiological evaluations. **(a)** Before treatment; yellow circle represents mediastinal lesion, yellow arrow represents primary lung tumor. **(b)** Efficacy evaluation was SD after 3 months of first-line aletinib treatment. **(c)** Disease progression after 4.5 months of aletinib treatment, the mediastinal lesion invaded bilateral main bronchus, and the left main bronchus was nearly occluded; red arrow represents intratracheal invasion. **(d)** First efficacy evaluation was SD after 1 month of second-line dual-target combination therapy. **(e)** Disease progression after 2 months of dual-targeted combination therapy with partial enlarged mediastinal lesion and a suspicious new lymph node metastasis in the right neck; red circle represents new lymph node metastasis. **(f)** The timeline of treatment. **(D)** Changes of tumor infiltration under bronchoscopy. **(a)** The mediastinal lesion invaded the carina and bilateral main bronchus after 4.5 months of first-line alectinib treatment. **(b)** The tumor infiltration was slightly relieved after 1 month of second-line dual-target combination therapy. **(E)** Dynamic monitoring of tumor markers during treatment. NGS, next-generation sequencing; SD, stable disease.

A 44-year-old non-smoking female was admitted to our hospital in June 2020 because of persistent cough. Positron emission computed tomography (PET-CT) revealed a mass in the right middle lobe and enlarged lymph node in the mediastinum. The mediastinal lesion surrounded the right main bronchus. She underwent ultrasonography-guided percutaneous right cervical lymph node biopsy. The pathologic examination showed poorly differentiated adenocarcinoma (cT1bN3M1a, stage IVA) (**Figure 1A**). A novel *IGR* (upstream *C2orf16*)*-ALK* exon20 (abundance: 9.7%) fusion was identified by a NGS assay based on 139-gene panel (GENESEEQ Technology Inc, Nanjing, China) (**Figure 1B**), and mutations of *TP53* (abundance: 42.6%) and *ERBB4* (abundance: 18.7%) were also identified in the same tumor tissue (see **Supplementary Material** for more detailed genetic informations). The patient first received continuous oral alectinib, 600 mg twice daily. The lung lesion continued to shrink, but the mediastinal lesion did not change significantly. Progression of the disease was observed 4.5 months later due to mediastinal lesion invading bilateral main bronchus (**Figures 1C, D** **)**, thus bronchus stents were placed to alleviate tracheal stenosis. To clarify the genetic alterations, a NGS analysis was recommended again after disease progression. However, the patient refused because of the high cost. We then re-evaluated the alterations of the coughed-up tissues by IHC, of note, IHC revealed positive expression of ALK-D5F3 and BRAF-V600E (**Figure 1A**). Considering that alectinib was already resistant and the fact that the third-generation ALK inhibitor lorlatinib was not accessible in China at that time, and that the patient herself was very resistant to chemotherapy out of fear of its adverse effects, she was subsequently treated with dabrafenib, 75 mg twice daily, plus trametinib, 2 mg once daily, combined with alectinib, 600 mg twice daily. The follow-up CT scan performed 1 month later showed stable disease (SD), and the tumor infiltration was slightly relieved under bronchoscopy (**Figures 1C, D** **)**. Considering the resistance of this patient to alectinib, and the fact that lorlatinib was not accessible, alectinib was then replaced with ceritinib, 450 mg daily, combined with dabrafenib and trametinib. Despite switching to second-line treatment, tumor markers increased sharply (**Figure 1E**) and progression of disease was observed again 2 months later (**Figure 1C**), the patient died shortly owing to rapid disease progression.

### Case 2

**Figure 2:**
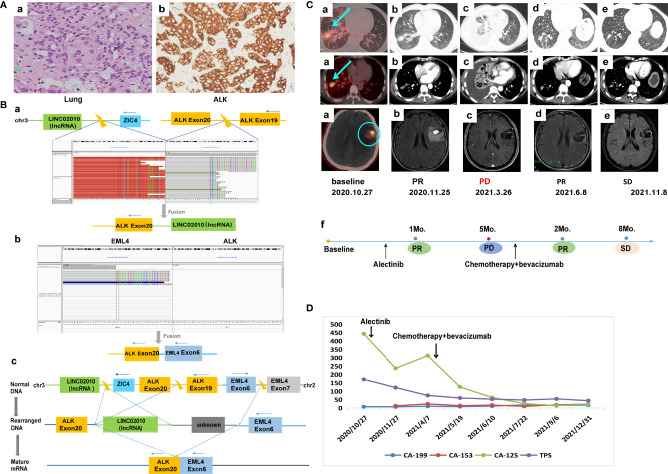
Case 2. **(A)** Pathology results. **(a)** H&E staining shows lung adenocarcinoma (40×). **(b)** IHC shows positive expression of ALK (40×). **(B)** Identification of the fusion sequence by NGS. **(a)** The identification of IGR-ALK by DNA sequencing, visualized by the Integrative Genomics Viewer (IGV). **(b)** The identification of EML4-ALK by RNA sequencing. **(c)** Schematic diagram illustrating molecular rearrangements that explain the emergence of the EML4 and ALK mRNA based on the DNA and RNA NGS results. **(C)** Radiological evaluations. **(a)** Before treatment; the blue arrow represents primary lung tumor, the blue circle represents intracranial metastasis, which maintained stable throughout the course of treatment. **(b)** First efficacy evaluation was PR after 1 month of first-line alectinib treatment. **(c)** Lung lesion progression after 5 months of first-line alectinib treatment **(d, e)** Continuing response to second-line chemotherapy in combination with bevacizumab. **(f)** The timeline of treatment. **(D)** Dynamic monitoring of tumor markers during treatment. NGS, next-generation sequencing; PR, partial response.

A 55-year-old Chinese male with no history of smoking or alcohol consumption visited our hospital on 24 October, 2020 with chief complaints of discovering lung occupation and aphasia for 4 days. PET-CT revealed a mass in the right lower lobe as well as pleural effusion, with bilateral pulmonary, multiple lymph node, bone, adrenal gland, and brain metastases. A pathologic diagnosis of lung adenocarcinoma was established by percutaneous needle biopsy of the left supraclavicular lymph node (cT4N3M1c, stage IVB) (**Figure 2A**). IHC revealed positive expression of ALK-D5F3 (**Figure 2A**). A rare *IGR* (downstream *ZIC4*)*–ALK* exon20 (abundance: 22.6%) fusion was found by a NGS assay based on 425-gene panel (GENESEEQ Technology Inc, Nanjing, China) (**Figure 2B**), the other mutations, including *CDKN1C*, *CYP2A6*, *FLT4*, *LRP1B*, *MAP2K4*, *PTK2*, *ROS1*, *KDR*, *TP53*, were also been detected in the same tumor tissue (see **Supplementary Material** for more detailed genetic informations). The patient consented to take alectinib at 600 mg orally twice daily, in combination with intracranial gamma knife therapy for brain metastases. Five months later, the efficacy evaluation was progress disease (PD) due to progression of lung lesions (**Figure 2C**). To clarify the genetic alterations after alectinib resistance, a NGS assay based on a 139-gene panel (GENESEEQ Technology Inc, Nanjing, China) was performed on the cervical lymph node biopsy tissue, and no *ALK* fusion was detected. In the light of the NGS result and clinical guidelines, he received six cycles of PC regimen (pemetrexed + carboplatin) chemotherapy plus bevacizumab anti-angiogenic therapy, and continued to receive pemetrexed and bevacizumab as maintenance therapy. Tumor markers dropped significantly. The patient’s best response was partial response (PR) and maintained SD during the treatment, with intracranial metastasis maintained stable throughout the course of treatment (**Figures 2C, D** **)**, achieving a PFS of over 1 year up to the last follow-up assessment.

## Discussion

At present, more than 20 intergenic ALK fusion genes have been reported, and only a few of these are sensitive to ALK-TKIs, including *CENPA/DPYSL5*, *CENPA*, and *CHRNA7* (6, 7). Therefore, comprehensive molecular analysis of the different ALK fusion partners is crucial to guide clinical precision treatment. In this study, we identified two novel *IGR-ALK* fusions at the DNA level which manifest *EML4-ALK* fusions at the RNA level. The breakpoints were located in the intergenic region between *GCKR* and *C2orf16* in case 1, and between *LINC02010* and *ZIC4* in case 2, and exon20 of the *ALK* gene. The kinase domain was retained in *ALK* exon20 and fused with the *IGR* to form *IGR-ALK*. It was speculated that the *IGR* fused with *ALK* and the unknown region fused with *EML4* were removed during transcription, resulting in the formation of mature mRNA, which was consequently detected as *EML4-ALK*. Intergenic-breakpoint fusions, in which genomic breakpoints are located in intergenic regions of DNA, where there is no genetic effect, are theoretically unlikely to produce chimeric fusion protein as there are no chimeric full-coding transcripts. However, Davies, Kurtis D et al. point out that genomic breakpoint determined by DNA sequencing is an unreliable predictor of breakpoint at the transcript level (8). Thus, whether these intergenic-breakpoint fusions can be activated remains elusive, and how they affect the response to targeted therapy remains unclear.

There are several molecular tools available that can detect carcinogenic ALK fusions at different levels. Fluorescence *in situ* hybridization (FISH) can detect gene fusions at the DNA level and has been considered the gold standard for ALK gene rearrangements, but it can produce false-positive results owing to unproductive rearrangements (9). IHC can be used as a primary screening tool with the characteristics of time-saving and economical, but most IHC assays detect from the protein level, not the fusion epitope, thus are not able to identify the nature of the fusion variant (9). Reverse transcription-polymerase chain reaction (RT-PCR) is also a common approach to detect rearrangement of certain variant with the advantage of being highly sensitive, however, it can only detect certain types of fusion identified by commercial PCR kits. Compared with traditional detection techniques, NGS can simutaneously detect multiple gene fusions and identify fusion partners and breakpoints, helping to elucidate the molecular mechanisms behind complex gene rearrangement events (9).

The global randomized phase III ALEX trial demonstrated that alectinib was significantly superior to crizotinib in terms of efficacy and toxicity in untreated ALK-positive NSCLC, establishing alectinib as the new standard first-line treatment (10). However, there remains a lack of treatment recommendations for rare ALK fusions. We referred to the current first-line treatment for ALK-positive NSCLC, unfortunately, both our patients showed limited response to alectinib unlike conventional *EML4-ALK* fusion that responded well to matched targeted therapy. Some studies have demonstrated that the fusion partners can affect the response to ALK-TKIs (11–13); specifically, different variants exhibit different protein stabilities, which in turn affect sensitivity to ALK-TKIs *in vitro* and lead to numerically different median PFS durations *in vivo* (11). The mechanism of these two novel *IGR*-*ALK* fusions regarding resistance to alectinib is unknown and needs further investigation.

After first-line ALK-TKI resistance, mutations that dependent on ALK signaling may still benefit from sequential ALK-TKIs treatment (14), for ALK “off-target” patients, whose driver mutation is based on a different mechanism, posterior-line treatment options are limited. Immunotherapy has surpassed chemotherapy as the first choice for second-line treatment (15–19), however, the results of immunotherapy are disappointing in patients with ALK mutation (15–19). Our previous study also confirmed the transformation of the tumor microenvironment to immunosuppressive phenotype after TKI resistance (20). Bevacizumab is an anti-vascular endothelial growth factor (VEGF) monoclonal antibody that can degrade the existing tumor vascular system, inhibit the growth of new blood vessels and resist vascular permeability, thus controlling tumor growth (21–23). Studies have shown that the addition of bevacizumab significantly improves the clinical benefit of advanced non-squamous NSCLC compared to platinum-based chemotherapy (24–26). In addition, bevacizumab was also shown to be effective in brain metastases in the BRAIN study (27). In the light of the NGS result and clinical guidelines (CSCO Guidelines), patient 2 received chemotherapy combined with bevacizumab as a second-line treatment strategy after failure of first-line alectinib treatment. Surprisingly, the second-line treatment yielded encouraging results, suggesting that chemotherapy combined with bevacizumab may be a more preferable treatment strategy than alectinib for these two types of novel *IGR-ALK* fusions. Therefore, it may be more appropriate to choose chemotherapy combined with bevacizumab directly rather than ALK inhibitors when treating these two novel *IGR-ALK* fusions in patients with NSCLC. Further clinical studies are needed to verify our findings.

Based on literature retrieval, this is the first report to demonstrate the presence of a novel *C2orf16*-*ALK* fusion and a novel *LINC02010-ALK* fusion in NSCLC. Although manifested to be *EML4-ALK* fusions by RNA sequencing, both our patients showed limited response to alectinib unlike conventional *EML4-ALK* fusion that responded well to matched targeted therapy, suggesting that these two novel *IGR*-*ALK* fusions may not be sensitive to conventional alectinib treatment. In summary, the value of our study lies in the finding of two novel *IGR*-*ALK* fusions which were not effective against conventional alectinib therapy and in the proposal of a more preferable therapeutic strategy (chemotherapy combined with bevacizumab therapy), and we further emphasize the need for comprehensive molecular analysis of different ALK fusion partners at the DNA level to formulate accurate treatment strategies.

## Data availability statement

The original contributions presented in the study are included in the article/**Supplementary Material**. Further inquiries can be directed to the corresponding author.

## Ethics statement

Written informed consent was obtained from the participant for the publication of this case report.

## Author contributions

WL and YW contributed to the planning and supervision of the study. SL contributed to data curation, visualization and drafted the manuscript. HS and JW contributed to manuscript revision. HL contributed to the review of clinical data. YF contributed to the literature research and discussion. All authors contributed to the article and approved the submitted version.

## Conflict of interest

The authors declare that the research was conducted in the absence of any commercial or financial relationships that could be construed as a potential conflict of interest.

## Publisher’s note

All claims expressed in this article are solely those of the authors and do not necessarily represent those of their affiliated organizations, or those of the publisher, the editors and the reviewers. Any product that may be evaluated in this article, or claim that may be made by its manufacturer, is not guaranteed or endorsed by the publisher.

## Abbreviations

ALK, anaplastic lymphoma kinase; EML4, echinoderm microtubule-associated protein-like 4; FISH, fluorescence *in situ* hybridization; IGR, intergenic region; IHC, immunohistochemistry; NGS, next-generation sequencing; NSCLC, non-small cell lung cancer; PD, progress disease; PET-CT, positron emission computed tomography; PFS, progression-free survival; PR, partial response; SD, stable disease; RT-PCR, reverse transcription-polymerase chain reaction; TKIs, tyrosine kinase inhibitors; VEGF, vascular endothelial growth factor.
