# Supplementary material for: Case report: Two novel intergenic region-ALK fusions in non-small-cell lung cancer resistant to alectinib: A report of two cases
Source: Front Oncol. 2022 Jul 22;12:916315. doi: 10.3389/fonc.2022.916315 (PMC9356229; doi:10.3389/fonc.2022.916315)
Supplement: Supplementary file 1 [file DataSheet_1.docx]

**Next-Generation Sequencing**

**DNA-based NGS**

The patients were evaluated using 425 or 139 gene NGS panel in a centralized clinical testing center (Nanjing Geneseeq Technology Inc.) according to protocols reviewed and approved by the ethical committee of NanFang Hospital, Southern Medical University (Guangdong, China). DNA extraction, sequencing library preparation, and targeted capture enrichment were carried out following the methods as previously described with modifications (1). Target enriched libraries were sequenced on the HiSeq4000 platform (Illumina). Single-nucleotide variants, indels, structural variants, and copy number changes were identified by validated bioinformatics process from parafﬁn-embedded tissues (2-5). Mutant allele frequency (MAF) cutoff for single-nucleotide variants and indels was defined as 1%. The log2 ratio cut-off for copy number gain was defined as 2.0 for tissue samples. A log2 ratio cut-off of 0.6 was used for copy number loss detection.

**RNA-based NGS**

RNA extraction

Total RNA from cultured cells was extracted with PureLink/Trizol according to the manufacturer’s instructions. The amount, quality and composition of isolated RNA were analysed by Nanodrop, Qubit 3.0 for total RNA and an Agilent 2100 Bioanalyzer for total RNA integrity. RNA purity was checked using the NanoDrop One/One C (Thermo Fisher Scientific, CA, USA). RNA concentration was measured usving Qubit RNA HS Assay Kit in Qubit3.0 Flurometer (Life Technologies, CA, USA). RNA integrity was assessed using the Agilent RNA 6000 Pico Kit of the Agilent Bioanalyzer 2100 system (Agilent Technologies, CA, USA).

RNA-seq

RNA-sequencing was performed by Nanjing Geneseeq Inc., Nanjing, China. Sequencing libraries were generated using KAPA Stranded RNA-Seq Kit(KAPA, USA) following manufacturer’s recommendations and index codes were added to attribute sequences to each sample. Libraries were pooled and sequenced on an Illumina X-ten PE150 platform. A minimum of 6G data were generated per sample.

**REFERENCES**

1. Tong L, Ding N, Tong X, Li J, Zhang Y, Wang X, et al. Tumor-derived DNA from pleural effusion supernatant as a promising alternative to tumor tissue in genomic profiling of advanced lung cancer. *Theranostics*. (2019) 9: 5532-41. doi: 10.7150/thno.34070

2. DePristo MA, Banks E, Poplin R, Garimella KV, Maguire JR, Hartl C, et al. A framework for variation discovery and genotyping using next-generation DNA sequencing data. *Nat Genet*. (2011) 43: 491-8. doi: 10.1038/ng.806

3. Newman AM, Bratman SV, Stehr H, Lee LJ, Liu CL, Diehn M, et al. FACTERA: A practical method for the discovery of genomic rearrangements at breakpoint resolution. *Bioinformatics*. (2014) 30: 3390-3. doi: 10.1093/bioinformatics/btu549

4. Amarasinghe KC, Li J, Halgamuge SK. CoNVEX: Copy number variation estimation in exome sequencing data using HMM. *Bmc Bioinformatics*. (2013) 14 Suppl 2: S2. doi: 10.1186/1471-2105-14-S2-S2

5. Shen R, Seshan VE. FACETS: Allele-specific copy number and clonal heterogeneity analysis tool for high-throughput DNA sequencing. *Nucleic Acids Res*. (2016) 44: e131. doi: 10.1093/nar/gkw520
